# Sulfitolytic and keratinolytic potential of *Chryseobacterium* sp. RBT revealed hydrolysis of melanin containing feathers

**DOI:** 10.1007/s13205-016-0464-0

**Published:** 2016-06-23

**Authors:** Ranjit G. Gurav, Jingchun Tang, Jyoti P. Jadhav

**Affiliations:** 1 Department of Biotechnology, Shivaji University, Kolhapur, 416004 India; 2 College of Environmental Science and Engineering, Nankai University, Tianjin, 300071 China

**Keywords:** 5,5′-Dithio-bis-(2-nitrobenzoicacid), *Chryseobacterium*, Dehairing, Keratinase, Melanin

## Abstract

In black feathers, melanin is embedded in keratin matrix that makes feather more resistance to the microbial degradation. *Chryseobacterium* sp. RBT previously isolated from the poultry waste disposable site revealed strong sulfitolytic and keratinolytic activities. Maximum keratinase activity was observed at 48 h (89.12 U ml^−1^) showed 83 % of native black feather degradation. The concentration of free sulfhydryl groups released during degradation was 0.648 × 10^−4^ M (12 h), 2.144 × 10^−4^ M (96 h), and however, declined on prolong incubation to 1.752 × 10^−4^ M (120 h). Melanin was released in the degradation medium after microbial exploitation of black feather. After purification, melanin was dark brown colored powder insoluble in water, 5 M HCL, ethanol, methanol, benzene, chloroform, and acetone; whereas, soluble in KOH and NaOH. On exposure to oxidizing and reducing reagents feather melanin showed decolorization, while formed a brown precipitate when reacted with FeCl_3_. The spectroscopic characterization of isolated melanin demonstrated absorption at infra-red region. Similarly, UV–visible scan confirmed that increase in the wavelength progressively declined the absorbance of pigment. The crude keratinase enzyme (2 % *v/v*) produced during degradation showed complete dehairing of goat skin within 20 h.

## Introduction

In birds, feather pigmentation mainly depends on the components, such as keratin and melanin, having different refractive indexes. Melanin is widely distributed in plants, animals, and some microbes, although it has been not assigned any specific role in the growth and development but to enhance their survival and competitiveness (Alois and Michael 1986). Chemically melanin was a dark colored, negatively charged, and high molecular weight heterogeneous polymer which was insoluble in most of the aqueous or organic solvents (Nicolaus et al. 1964; Crippa et al. 1989). Melanin is embedded in the β-keratin matrix arranged in a complex array of feather barbules (Durrer 1986). Besides pigmentation, melanin provides structural rigidity to feathers by cross-linking with proteins and making them more resistant against feather feeding microorganisms and ecto-parasites (Burtt 1986; Goldstein et al. 2004; Gunderson et al. 2008). Wilson et al. (2007) in their study with archaeological contexts found that microbial activity degraded only keratin without affecting the melanin. Similarly, Goldstein et al. (2004) and Gunderson et al. (2008) demonstrated the degradation of melanized feathers using *Bacillus licheniformi* and *B. licheniformi* OWU 138B and concluded that melanized feathers where unable to biodegrade.

Keratins are the highly specialized fibrous proteins ubiquitous in animals. Feather contains about 90 % keratin protein rich in disulfide bridges which are responsible for making feathers more resistant against common proteases, such as trypsin, pepsin, and papain, thus reduces the degradation process in nature (Onifade et al. 1998). However, some biological entities, such as insects (clothes moth larvae, carpet beetles, and chewing lice) and microorganisms (eucarya, bacteria, and archaea), can exploit the keratins. Microbes produce keratinases enzyme to breakdown the keratin and utilize it as a source of carbon, nitrogen, sulfur, and energy for its growth and development (Noval and Nickerson 1959). Previously, *Lysobacter* NCIMB 9497 (Allpress et al. 2002), *Microbacterium* sp. (Thys et al. 2004), *Xanthomonas maltophilia* (de Toni et al. 2002), *Stenotrophomonas* sp. (Yamamura et al. 2002), *Chryseobacterium* *indologenes* TKU014 (Wang et al. 2008), and *Serratia* sp. HPC 1383 (Khardenavis et al. 2009) were reported as keratin degraders.

Several researchers had isolated genus *Chryseobacterium* from the poultry carcasses and believed that this bacteria might have been originated from the poultry itself or from the slaughterhouse environment (Hang’ombe et al. 1999). Beer et al. (2005) stated that most of the *Chryseobacterium* were isolated from the raw chicken portion or whole bird at different stages of meat processing. Vandamme et al. (1994) have stated that genus *Chryseobacterium* was closely associated with strong proteolytic activity.

Therefore, in present study, previously isolated *Chryseobacterium* sp. RBT was demonstrated for the degradation of melanin containing native feathers that cannot be degraded by normal microbial flora. This was the first report signifying highest degradation of native black feather within short span of time. The biodegradation efficiency was analyzed by quantifying the keratinase enzyme, soluble protein, amino acid, and free sulfhydryl groups in the medium. During black feather degradation melanin embedded in the feather matrix was released in the medium. This melanin was purified and characterized which has many industrial appplications. Whereas, the crude keratinase enzyme was applied for ecofriendly dehairing of goat skin.

## Materials and methods

### Chemicals, black feathers, and goat skin

Standard melanin (synthetic) and 5,5′-dithio-bis (2-nitrobenzoic acid) were purchased from Sigma-Aldrich, USA. Keratin powder, bovine serum albumin, sodium hydroxide, hydrogen peroxide, ferric chloride, sodium hypochlorite, and potassium hydroxide were obtained from Himedia, India. Solvents, such as ethanol, methanol, chloroform, acetone, benzene, and phenol were procured from Sisco Research Laboratory (SRL), India. All the chemicals and reagents used were of the highest purity and analytical grade. Black feathers of slaughtered domestic fowl (*Gallus* *gallus domesticus*) collected from central chicken market, Kolhapur, India were washed, dried, and stored for further studies. Whereas, goat (*Capra hircus*-Osmanabadi breed) skin was obtained from the local meat market in Kolhapur, India.

### Microorganism and maintenance

The *Chryseobacterium* sp. RBT (genebank accession number GU481093) used in this study was previously isolated from the poultry waste contaminated site. Bacterial culture was routinely subculture and maintained on keratin agar containing (g l^−1^): Na_2_HPO_4_ (6.0), KH_2_PO_4_ (3), NaCl (5), MgSO_4_ (0.1), keratin powder (1), and agar (25) as a solidifying agent (Gurav and Jadhav 2013a).

### Biodegradation of black feathers

The black feather degradation experiment was carried out in basic salt medium (BSM) having composition (g l^−1^): Na_2_HPO_4_ (6.0), KH_2_PO_4_ (3.0), NaCl (5.0), MgSO_4_ (0.1), and native black feathers (10). This medium was inoculated with 0.1 % (v/v) of *Chryseobacterium* sp. RBT culture having absorbance 0.92 at 660 nm and incubated on the orbital shaker (140 rpm) at 37 °C (Gurav and Jadhav 2013a). The degradation rate was assessed by pulling aliquots at different time intervals to analyze the keratinase activity, free sulfhydryl groups, soluble proteins, and amino acids.

### Keratinase assay

The keratinase activity was determined using pure keratin powder as a substrate (Gurav and Jadhav 2013a). In brief, 1 ml of crude enzyme diluted in tris–HCl buffer and 1 ml substrate (0.1 % keratin powder) was incubated at 37 °C for 10 min. The reaction was terminated by adding 2 ml of 0.5 M trichloro acetic acid (TCA). This reaction mixture was centrifuged, and the absorbance was detected at 280 nm (UV-1800, Shimadzu, Japan). The enzyme control was treated in the same way except the TCA and was added before incubation. One unit (U ml^−1^) of keratinase activity was defined as an increase in corrected absorbance by 0.001 units at 280 nm with control min^−1^ (Cai et al. 2008) and calculated using formula:

$${\text{U}} = 4 \times n \times A_{280} /\left( {0.01 \times 10} \right),$$

where *n,* dilution rate; 4, final reaction volume; 10, incubation time (min).

### Quantification of free sulfhydryl groups

The quantification of free sulfhydryl groups released during feather degradation was executed using 5,5′-dithio-bis-(2-nitrobenzoic acid), also known as DTNB or Ellman’s reagent (Ellman 1959). Sulfhydryl groups in the degradation medium reacted with DTNB and produced a mixture of disulfide and 2-nitro-5-thiobenzoic acid (TNB) which was yellow-colored product measured at 412 nm. In brief, 200 μl of cell-free supernatant was added to 2.5 ml of reaction buffer (0.1 M sodium phosphate, pH 8.0, containing 1 mM EDTA) and 50 μl of DTNB solution (4 mg ml^−1^). This reaction mixture was incubated at room temperature for 15 min and absorbance was recorded at 412 nm (UV-1800, Shimadzu, Japan). Blank was prepared by adding 2.7 ml of reaction buffer and 50 μl of DTNB solution. The free sulfhydryl concentration was calculated using the following formula.

Molar absorptivity (*E*) is defined as follows:

$$E = A/bc,$$

where *A* is the absorbance; *b* is the path length in centimeters; *c* is the concentration in M l^−1^ (=M).

Solving for concentration gave the following formula:

$$c = A/bE.$$

The reported molar extinction coefficient of TNB in 0.1 M phosphate, pH 8.0, 1 mM EDTA was *E* = 14,150 M^−1^ cm^−1^ at 412 nm.

### Detection of soluble protein and amino acids

Soluble protein content of degradation medium was determined by folin-phenol method using BSA as standard (Lowry et al. 1951). Similarly, the amino acids were quantified by ninhydrin method using leucine as standard (Moore and Stein 1957).

### Percentage degradation of feather biomass

The percentage degradation of black feathers was determined on the basis of cell-free dry weight of the residual feathers in the culture broth at the end of experiment (Cortezi et al. 2008).

### Separation and purification of the melanin pigment

Melanin released in the feather degradation medium was filtered through the mesh to separate them from the undigested feathers. The filtrate contained the cell biomass and melanin was further centrifuged, and the resulted pellet was dissolved in NaOH to make the pH 12 and incubated at room temperature for 60 min. This mixture was then acidified by adding HCl to precipitate the pigment by decreasing the pH level to three (Sajjan et al. 2010; Surwase et al. 2013). After centrifugation, a dark blackish brown precipitate of crude melano-protein obtained was washed 4–5 times with doubled distilled water. This brown amorphous material was re-dissolved in 0.1 M NaOH and deproteinised with 20 % chloroform, and subsequently, the dark brown layer was then separated and re-precipitated with the acid. The obtained pellet was repeatedly washed with methanol and ethanol and was finally lyophilized. Chemical and spectroscopic characterizations were performed with reference to the synthetic standard melanin (Sigma, USA) (Surwase et al. 2013).

### Characterization of purified pigment

#### Qualitative chemical analysis

Chemical analysis of the purified pigment was done in parallel to the standard melanin. The pigment solubility was checked in distilled water, ethanol, methanol, chloroform, acetone, benzene, NaOH, and KOH. Similarly, the specific qualitative tests for melanin were performed using H_2_O_2_, FeCl_3,_ and NaOCl (Sajjan et al. 2010).

#### Spectral analysis

The purified pigment and standard melanin were dissolved in 0.1 M NaOH, and the UV–visible adsorption spectra were recorded between 220 and 800 nm (Shimadzu, Japan) using 0.1 M NaOH as blank. The purified feather melanin and standard melanin spectrum were plotted as optical density of compound against the wavelength (Surwase et al. 2013).

#### FT-IR study

Fourier transform infra-red spectroscopy (Perkin Elmer, Spectrum 100, USA) of the isolated pigment with reference to the standard melanin was done in the mid IR region of 400–4000 cm^−1^ with 16 scan speed. The pellet was prepared using spectroscopic grade KBr (10:90), and analysis was carried out and the changes in % transmission at different wavelengths were recorded (Sajjan et al. 2010).

### Enzymatic leather processing

The *Chryseobacterium* sp. RBT produced keratinase enzyme during black feather degradation was applied on goat skin to check the dehairing ability. Crude keratinase 2.0 % (v/v) was applied on clean goat skin placed in tris–HCl buffer (50 mM, pH 8.6) and kept at 37 °C (Gurav and Jadhav 2013a). The enzyme treated skin samples were washed thoroughly under tap water and visually observed for the dehairing, and data was recorded in the form of photographs and time required for skin dehairing.

### Statistical analysis

The data was analyzed by the one-way analysis of variance (ANOVA) with the Tukey–Kramer multiple comparisons test.

## Results and discussion

### Degradation of melanin containing feathers

**Fig. 1:**
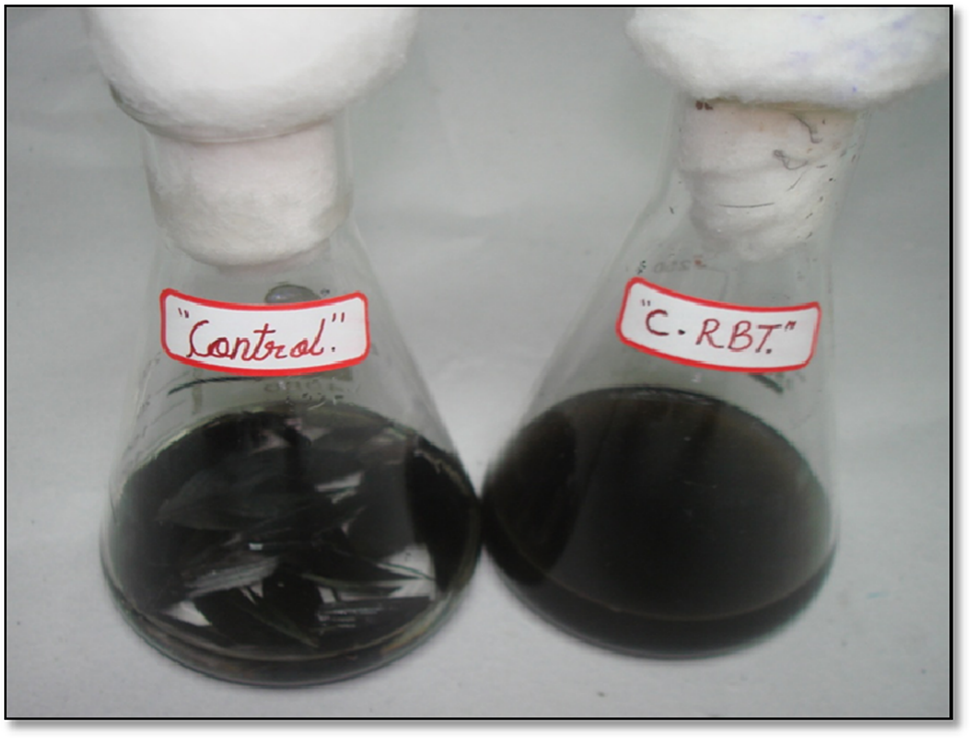
Degradation of native black feathers by releasing of melanin embedded in the feather matrix. ‘Control’ without inoculum and ‘C. RBT’ inoculated with *Chryseobacterium* sp. RBT (48 h)

**Fig. 2:**
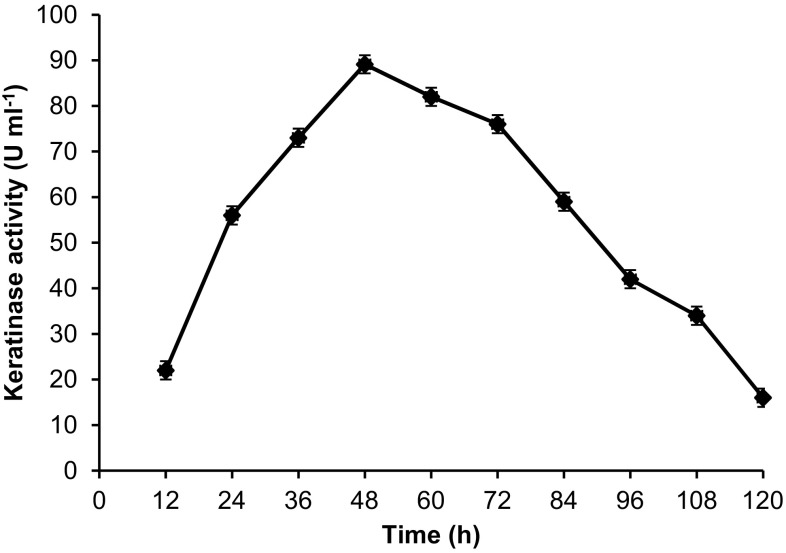
Keratinase activity (U ml^−1^) at different time intervals of feather degradation. Data shown as mean ± SEM, *n* = 3

In the present study, *Chryseobacterium* sp. RBT hydrolyzed the native black feathers of *Gallus gallus domesticus* and utilized it as sole source of carbon, nitrogen, and energy. The native black feathers were degraded (83 %) within 48 h, by releasing melanin embedded in the feather matrix (Fig. 1). Initially, the keratinase enzyme activity was observed at 12 h (21.9 U ml^−1^) and was highest at 48 h (89.12 ml^−1^). However, further incubation until 120 h (16.20 U ml^−1^) significantly declined the keratinase activity (Fig. 2). During the degradation process, an increase in the pH from 7.5 to 8.8 was observed. This tendency of medium alkalinity was attributed due to the deamination reactions leading to release of ammonia from protein, peptides, and amino acids. Such increase in pH during feather degradation is the important indication of high keratinolytic potential of the microorganisms (Riffel et al. 2003). Similarly, our previous study on poultry feather degradation using *Chryseobacterium* sp. RBT showed maximum keratinase activity at pH 8.6 (Gurav and Jadhav 2013a). On the other hand, medium alkalization may have facilitated the release of melanin embedded in the feather matrix, as it has been well documented by many authors that melanin pigment was likely to be solubilized at alkaline pH.

### Determination of free sulphydryl groups

**Fig. 3:**
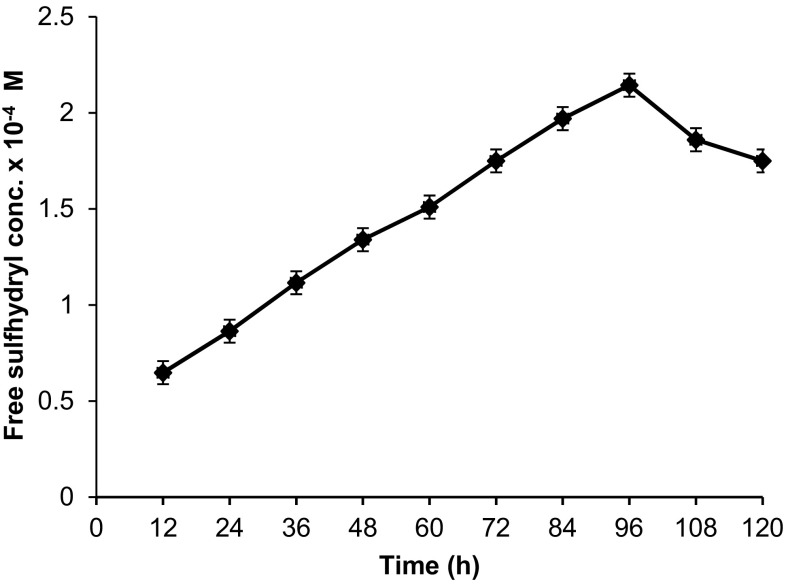
Analysis of free sulfhydryl groups released during feather degradation. Data shown as mean ± SEM, *n* = 3

Loss in the feather biomass was used as a primary and most reliable indicator for microbial keratinolytic abilities (Korniłłowicz-Kowalska 1997). However, other important indicators, such as peptides, amino acids, ammonia, sulfhydryl groups, sulfate excretion, keratinase activity, and medium alkalinisation, are also crucial to study the keratin degradation (Korniłłowicz-Kowalska 1997). Free sulfhydryl groups released during feather degradation were analyzed at different time intervals using 5,5′-dithio-bis (2-nitrobenzoic acid) (DTNB). DTNB reagent has high specificity for –SH groups at neutral pH, high molar extinction coefficient, and short reaction time. The concentration of free sulfhydryl groups in degradation medium was 0.648 × 10^−4^ and 2.144 × 10^−4^ M at 12 and 96 h, respectively. However, further incubation until 120 h reduced the content to 1.752 × 10^−4^ M (Fig. 3).

The cross-linking of protein chains by several disulphide bonds is the most distinctive character of keratins. Such bonding makes the keratin molecule more resistant to mechanical and enzymatic degradation. Many pathways were proposed to explain the microbial breakdown of keratin molecules amongst them one was cleavage of disulfide bonds prior to the proteolytic breakdown. This cleavage of the disulphide bond was carried out by sulfitolytic activities, which was determined by detecting free sulfhydryl groups in the culture media; these results were in agreement with the study of Onifade et al. (1998). The sulfitolytic cleavage of cystine was carried out by means of excess sulfite released by the microbial cells and was previously studied in filamentous keratinolytic fungi and actinomycetes; however, this pathway was also detected in bacterial degradation (Laba et al. 2015). Such disulphide bonds cleavage during microbial degradation was previously described in *Streptomyces pactum* (Böckle et al. 1995). Thus, in the present study, *Chryseobacterium* sp. RBT possesses a strong disulphide-bond breaking ability.

### Quantifying the peptides and amino acids

**Fig. 4:**
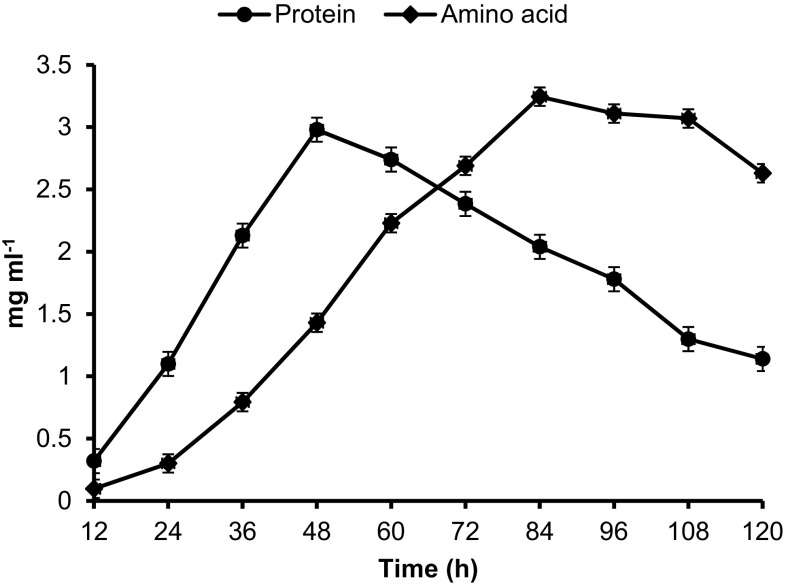
Protein and amino acid contents of degradation broth with time. Data shown as mean ± SEM, *n* = 3

The *Chryseobacterium* sp. RBT produces the keratinase enzyme responsible for the degradation of feather keratin into peptides and amino acids. Liberating the protein and amino acid during degradation served as a crucial indicator in feather keratin degradation. The highest soluble protein content was observed at 48 h (2.98 mg ml^−1^), whereas further incubation until 120 h declined the content to 1.14 mg ml^−1^ (Fig. 4). Similarly, the concentration of free amino acids was evaluated and was enhanced with time; this may be due to the peptides continued to breakdown into amino acids. The maximum free amino acids content was 3.24 mg ml^−1^ at 84 h of incubation (Fig. 4). A good correlation between protein, amino acids content, and feather biomass was observed during degradation.

In general, feather degrading microorganisms can either digest keratin through secretion of keratinases or otherwise grow directly on the feathers (Ruiz-Rodríguez et al. 2009). Microbial degradation of the feather biomass results into the hydrolysate rich in peptides, amino acids, and minerals, such as nitrogen, phosphorus, potassium, calcium, magnesium, iron, manganese, zinc, and copper (Gurav and Jadhav 2013b). Soil and foliar application of this feather hydrolysate to banana cultivation created a promising approach for nourishing the soil and also stimulated the production of banana fruits rich in health beneficial compounds, such as antioxidants (Gurav and Jadhav 2013b).

### Characterization of feather melanin

On purification obtained blackish brown colored pigment was insoluble in water, 5 M HCL, ethanol, methanol, benzene, chloroform, acetone, and soluble in KOH and NaOH. Whereas pigment showed decolorization after reacting with the oxidizing and reducing reagents, such as NaOCl and H_2_O_2_. Similarly, pigment produced a brown precipitate when reacted with FeCl_3._ All the results were compared and found identical with the synthetic standard melanin (Sigma).

### Spectroscopic analysis of the pigment

**Fig. 5:**
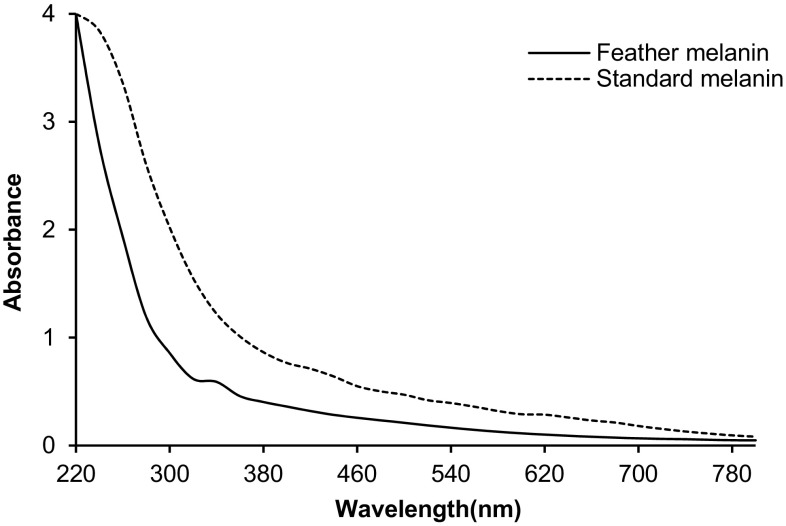
UV–visible spectral analysis of feather melanin (^___^) and standard melanin (^….^) pigment

The UV–visible absorption spectrum of synthetic and purified feather melanin is described in Fig. 5. The absorption was highest in UV region at 200–300 nm, but significantly declined towards the visible region, which was the characteristic property of melanin. This phenomenon was due to the presence of very complex conjugated arrangement in the melanin. A linear relationship between log absorbance and wavelength from 400 to 800 nm was observed, which was one of the most important criteria in melanin characterization. Such distinctive straight line with negative slope was demonstrated earlier in melanin produced by microorganisms (Sajjan et al. 2010; Surwase et al. 2013).

**Fig. 6:**
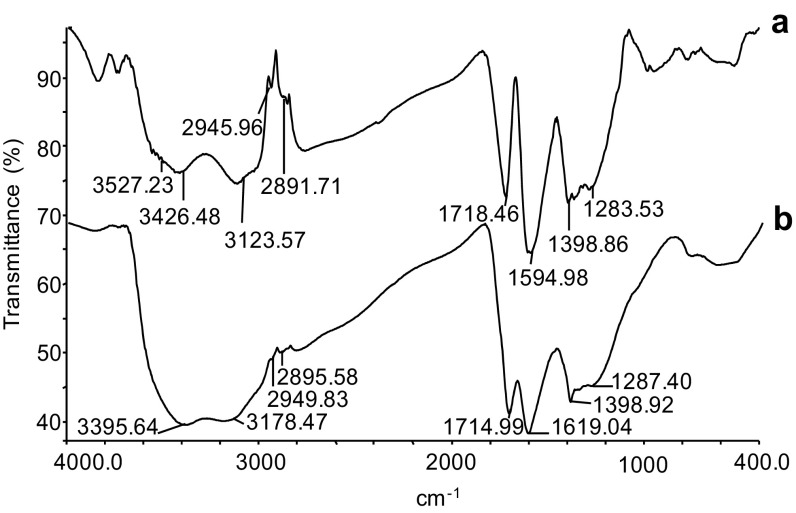
FT-IR spectrum of the melanin pigments: **a** synthetic melanin, **b** feather melanin

The FT-IR spectroscopy was preferred for additional characterization of the feather pigment. FT-IR was the most revealing, well-resolved, and non-destructive method, providing information on functional groups and detailed structural analysis (Sajjan et al. 2010; Surwase et al. 2013). The IR spectrum of feather melanin and standard melanin showed high degree of similarity with each other (Fig. 6). This characteristic property of the IR spectrum of melanin pigment was analogous with many earlier reports.

The obtained feather melanin could be used as an ingredient in photo protective creams, sunscreen lotions, bioinsecticidal preparations, removal of the heavy metals found in the environment, and protective against reactive oxygen and reactive nitrogen species (Sajjan et al. 2010; Surwase et al. 2013).

### Leather dehairing

**Fig. 7:**
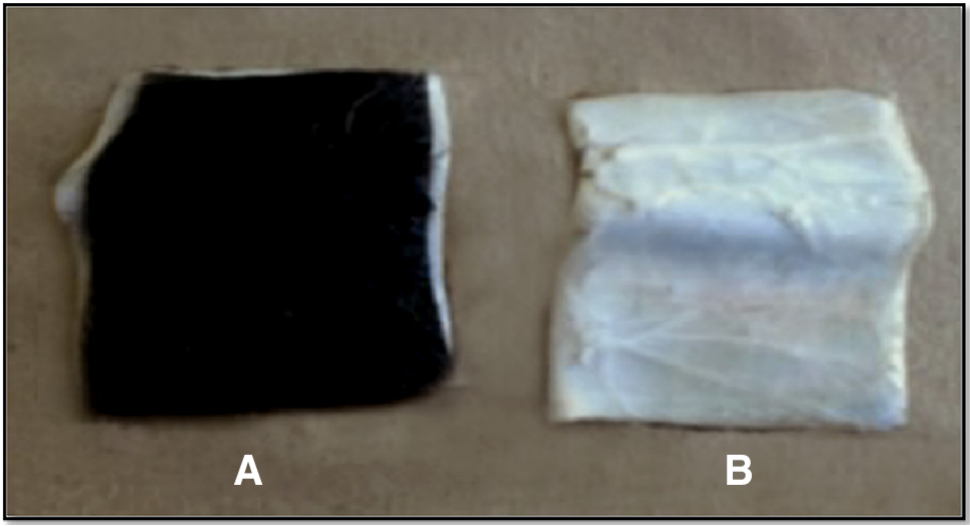
Enzymatic dehairing of goat skin: **a** without treatment, **b** crude enzyme treated (20 h)

The crude keratinase enzyme after applying on goat skin showed effective dehairing within 20 h (Fig. 7). Mechanical forces were not needed in enzymatic treated skin to remove the hair. However, the untreated goat skin showed intact hair with no hair releasing signs even by plucking with the forceps.

Currently, lime and sodium sulphide is widely applied for dehairing of the skin however; such chemicals are hazardous to humans and the environment (Malathi and Chakraborty 1991). In the enzymatic dehairing process, a selective breakdown of keratin tissue in hair follicle was observed which releases the intact hair without affecting the tensile strength of leather and the removed hair can be further used for vale addition (Macedo et al. 2005).

## Conclusion

*Chryseobacterium* sp. RBT degraded the native black feathers within short span of time liberating melanin embedded in the feathers matrix. This bacterium exhibited both keratinolytic as well as sulfitolysis activities that facilitated the breakdown of protein and di-sulphide bonds in keratin. The isolated feather melanin showed similar physical and chemical properties with synthetic melanin which could be exploited for many industrial applications. Whereas the crude keratinase enzyme could be employed as an effective and ecofriendly alternative in leather processing. Results of this research work can guide and lead to the anticipated path for biotechnological interventions to produce the value added products from the waste feather biomass.
